# The American Medical College Association

**Published:** 1880-10

**Authors:** 


### Editorial.

---

### THE AMERICAN MEDICAL COLLEGE ASSOCIATION.

Since the last annual meeting of the above named association, which was held in New York about the first of June, 1880, its position in relation to the progress of medical education and its prospects for the future, have been commented on by many of the medical journals in different parts of the country. These comments related chiefly to the action of the association in relation to the colleges requiring attendance on three regular annual courses of instruction before graduation, and to the withdrawal of certain Eastern schools from membership. The adoption of the amendment to the articles of confederation by which all the college members become pledged, not only to exact three years of professional study, but also attendance on three regular annual courses of college instruction before graduation, to become operative in 1882, was a very important step; and appears to have elicited only commendatory notices from the medical press.

It is true that the *St. Louis Clinical Record* offered some criticisms. They were not against the movement itself, however, but simply against the postponement of its practical operation for *two years.* The voluntary withdrawal from membership in the association, of the two most influential medical colleges in New York, and the intimated withdrawal of the Jefferson School in Philadelphia by its representative in the last meeting, thereby leaving the membership of the association almost wholly west of the Alleghanies and within the great interior valley of the continent, has evidently taken many of those connected with the medical press by surprise, and has led to much inquiry for the reasons actuating the withdrawing colleges. When it is remembered that the representatives from the College of Physicians and Surgeons, and the Bellevue Hospital Medical College (the two New York schools recently withdrawn), were among the most active in originating the association and in framing its constitution and articles of confederation, it is not surprising that their withdrawal before the completion of the fourth year of its progress, should create not only surprise in the minds of others, but much curiosity to know the reasons for such capricious action. Indeed, it is difficult to see how the colleges concerned could consistently or honorably withdraw without assigning true and adequate reasons for such a step. Yet we do not learn that any reasons whatever have been given by either of the colleges alluded to. But while the withdrawing colleges are themselves silent in regard to reasons for their action, *The Medical Record of New York* for August 14th, attempts to give some very singular reasons for them. After admitting that the association had "certainly done some good work;" that its claims to having secured progress as regards medical education in several important directions, "are to some extent just;" and finally, that it "has had the courage to take the step of insisting upon *three* full courses of lectures as a requisite for graduation;" the editor with apparent reluctance adds:

"But in spite of all this evidence of earnest endeavor after reform, we find that none of the *largest* medical colleges are working with the association. Not one of the colleges of Philadelphia, New York, or Boston, were represented at the meeting, [in New York] except the Jefferson Medical College, and its delegate *failed to vote* for the resolution requiring a three-term course. The College of Physicians and Surgeons of this city, [New York] and the Bellevue Hospital Medical College, withdrew from the association altogether. It is composed, therefore, now, for the most part, of the smaller medical colleges *scattered* throughout the West and South. This disaffection of the Eastern colleges, has, we fear, an unfortunate significance. If we interpret it rightly, it means that the association can do them no good, and that they do not have faith in the possibility of securing any great reforms in medical education through its influence. There certainly is some reason for looking at the matter in this light. The association is composed largely of colleges which really cannot subscribe to certain most desirable measures without ceasing to exist. One of these reforms—one perhaps of least importance—is that of raising the lecture fees. An attempt to make a rule on this point at the last meeting failed utterly. There is very sharp competition among some colleges, and one form which this takes, is the practice of underbidding each other in the matter of fees. This practice which is very demoralizing, the association cannot stop. Another reform which is greatly needed is the insisting that a medical college should furnish a certain amount of clinical instruction. But there are a good many country colleges, where a rule enforcing even a small minimum could not be carried out. The fact is, therefore, that a high minimum of requirements for graduation—a minimum embracing all points desirable to secure a thorough medical training—can never be applied to all American colleges, and can never be adopted by the college association without a fatal loss of membership. A minimum that would perhaps elevate and benefit small colleges would be inadequate for the institutions of the great cities."

There are several points in the foregoing quotation, from one of the most candid and enlightened of our contemporaries on the Atlantic border of our country, worthy of notice. The first is the very complaisant manner of assuming that all the largest medical schools in the country are centered in Philadelphia, New York and Boston; and as these have ceased to act with the American Medical College Association, the latter is now composed for the most part of "the smaller medical colleges scattered throughout the West and South." This kind of assumption of superior importance—greater size—and more perfect development of everything on our Atlantic borders, is characteristic of Eastern writers and talkers. They so habitually speak and write of the schools, colleges and cities of the West and South as "provincial," "small," "scattered," etc., as compared with the metropolitan character of those in the East, that we have often wondered whether they have ever studied the geography of their country, or cast a glance over the census statistics of the last two decades. Is it possible that the editor of the *Medical*

*Record* has not yet learned that a large majority of the people of the United States live west and south of the Alleghany mountains—that within the Mississippi valley alone, there are four or five cities each containing from a quarter to over half a million of inhabitants, with schools, medical colleges and hospitals, that will not suffer by the most rigid comparison with those in the largest cities in the East, and, moreover, that all these medical colleges are still members of the American Medical College Association? The truth is that the oldest medical college in Chicago, which was founded less than forty years since, has a class of students much larger than that of either the Pennsylvania University or the Harvard College at Boston, and only a little less than that of either the College of Physicians and Surgeons or the Bellevue College in New York, or of the Jefferson College in Philadelphia; and the aggregate number of medical students annually attending the medical colleges in Chicago, Cincinnati, St. Louis, Louisville and New Orleans, is very nearly equal to the aggregate of those attending the medical colleges of Philadelphia, New York, Brooklyn and Boston. And even if our Eastern confréré should turn his attention to the medical colleges in the smaller towns and cities "scattered throughout the West and South," could he find *one*, even in the most remote frontier settlement, with a smaller class in attendance than that which annually assembles in the medical department of old Yale, or with an annual college term of less than sixteen weeks, like that of Dartmouth and others in New England?

The next point worthy of notice in the foregoing extract from the *Medical Record* relates directly to the reason assigned for the withdrawal of the Eastern colleges, namely: "that the association can do them no good and that they do not have faith in the possibility of securing any great reforms in medical education through its influence." Without stopping to comment on the purely selfish motive implied in the first line of this quotation, let us try to find out what "great reforms in medical education" the writer and the colleges for which he speaks have in mind, and then we can more readily see whether the lack of "faith" spoken of is justified by anything in the past history of the association or not. The reader will see that the only measures of "great reform" actually mentioned in the *Record* are: first, "that of raising the lecture fees," and second, "the insisting that a medical college should furnish a certain amount of clinical instruction." In regard to the first, he says an attempt to make a rule on the subject of fees at the last meeting of the association "failed utterly." In looking over the records of the last meeting as published by the secretary, the only allusion we find to any attempted action on this subject is in these words: "On motion, it was resolved that, in the judgment of this association, the minimum lecture fees should be seventy-five dollars." How the adoption of such a resolution, expressly stating what ought to be the *minimum* for lecture fees, constituted an utter failure we leave for the *Record* to explain. But what has been the influence of the association over this subject of fees?

At the time of completing the organization of the association in 1877, with the exception of two colleges in St. Louis and one in New Orleans there were no medical colleges in the interior valley from the Great Lakes to the Gulf of Mexico whose lecture fees did not range between twenty and sixty dollars; several gave two graduating courses each year, and at Louisville there was a college faculty running a medical college under two names, the term of one following the other so as to enable any student to attend his *two* college courses and graduate in *nine months*, and at the same time inviting students by circulars sent into every Congressional district in the country to come on pretended free scholarships, paying only some nominal incidental fee instead of regular lecture fees. Starting with this state of things, the organization of these colleges into the American Medical College Association, with the resulting personal contact of their several representatives, their better acquaintance, clearer perception of mutual interests and greater confidence in each other, in the three short years that have elapsed, the double headed nuisance at Louisville has been completely broken up; not a single college continues to give two courses counting for graduation in one year, and every college of note or material influence, except the Medical Departments of the State Universities of Michigan and Iowa, from Detroit to New Orleans, has increased its lecture fees to seventy-five dollars. Could the editor of the *Record* or any other party reasonably ask for better progress in the same time, so far at least as concerns the colleges of the West and South? And yet not a college in either of these sections has withdrawn from the association on account of this advance on the subject of lecture fees. Is it not possible, however, that if the editor of the *Record* would make thorough search into the relations of things nearer home, he could find something relating to *fees* that might have had an influence on the action of the New York colleges? The double headed Kentucky-Louisville college "out West," was not the only college in the United States that sought to gather in large classes by issuing an undefined number of scholarships and liberal provisions for beneficiaries, if the signs of the times and the annual announcements do not lie, one of the *largest* colleges in the *largest* city in the East, called the Medical Department of the New York University, is in the habit of making both these methods available to a very liberal extent. The character of the very first *topic* taken up for consideration in the convention of college delegates held in Philadelphia in 1876, as well as the stringent nature of some of the articles of confederation relating to the same topic, adopted at the meeting in 1877, taken in connection with the active part performed in framing those articles by the representatives of the College of Physicians and Surgeons of New York, and of the Bellevue College, justifies, at least, the suspicion that a strong, underlying motive for the movement was a hope or expectation that the New York University would be drawn into the membership of the Association and thereby forced to abandon its plausable, indirect underbidding processes or suffer open disgrace. And is it not just possible that the failure of the last named institution to come into the organization, by which it maintained its own freedom while its immediate neighbors had placed themselves under additional restrictions, was really the efficient cause of their speedy loss of *faith* and their hasty retreat from an organization they had been so active in forming? We wish to call the attention of our brother of the *Medical Record* to another item closely related to the foregoing. All of the *smaller* medical colleges of the country are not "scattered throughout the West and South;" but there are still quite a number in the old States of Vermont, New Hampshire, Maine and

Connecticut, and there has long existed a relation between some of these schools and those of the great cities of the East by which facilities for the graduation of students were afforded almost equal to that of the former Kentucky-Louisville institution.

For instance, the medical department of Dartmouth, New Hampshire, has a short annual term of from fourteen to sixteen weeks, commencing early enough in the season to end about the time that the schools in Boston and New York commence, so that the student can take his first course there and his second course and graduate in one of the great cities, by an almost continuous college attendance of about *nine* months. Or he can go to the Long Island Hospital Medical College in the *great* city of Brooklyn, and attend its course commencing in the spring, and to one of the great schools in New York or Philadelphia the following autumn and graduate, having completed his *two courses* of college attendance all in twelve months. This nice arrangement for facilitating the making of doctors was blocked so far as the members of the American Medical College Association is concerned by the article of confederation requiring the lapse of, at least, fifteen months between the beginning of the first and the end of the second course of lectures the student is required to attend. Another of the articles of confederation bound the members of the association to require of each candidate for graduation evidence of having studied medicine *three full* years, which was designed to stop the common practice long followed in the larger colleges in the great Eastern cities, of graduating students who had attended two courses of lectures, but who had not studied medicine a day over eighteen months. We ask again, if it is not possible that finding themselves likely to be restricted by these wholesome regulations while their immediate neighbors, the New York University, the Harvard College, etc., remained free, had much to do in helping to dissipate their *faith* in the benefits to be derived from the association?

If, after due investigation, our New York confrère should find these suspicions to which we have given utterance, well founded, we hope he and others will hereafter cease to make the smaller medical schools "scattered throughout the West and South" the scape-goats for all the sins committed under the head of medical education. The second reform mentioned by the *Record* as desirable, but which it is alleged the members of the college association could not carry out, on account of the numerous smaller medical colleges scattered throughout the country, was that of providing adequate *clinical* instruction as a part of their regular system of teaching. We admit that there are a considerable number of small medical colleges both in the East and West (quite as many in proportion to the population in the former as in the latter), that have no adequate means for clinical instruction. But this, instead of furnishing an excuse for withdrawing, or standing aloof from membership in the college association, is one of the strongest reasons why such an association should be sustained. And if the old and well established medical colleges in the great Eastern cities, Baltimore, Philadelphia, New York, Brooklyn, and Boston, would at once enter the college association and unite with the colleges that are already members, in Detroit, Chicago, Cincinnati, Louisville, St. Louis, and New Orleans, not only in honestly maintaining the steps in advance already taken by the association, but in adding two others, namely, a just and proper standard of preliminary education on the part of students, and the furnishing of adequate *clinical instruction* in hospitals sufficient for the purpose, they would not only give it efficiency and permanence, but they would also cause it to be sustained by the whole body of the profession, including the county, State, and national medical societies, together with the State and national boards of health and boards of examiners. The only members that would be lost to the association by such a step, would be such as ought to be lost to the world by ceasing to exist, and whose loss would be "*fatal*" to no one but themselves. This part of the subject, however, is of such paramount importance as to require thorough discussion; but we have already occupied so much space that we must defer further comments until the November number.

